# Atypical Case of Presumed *Bartonella henselae* Osteomyelitis in a Child with Cat-Scratch Disease Mimicking a Malignant Bone Tumor

**DOI:** 10.3390/children13091126

**Published:** 2026-08-23

**Authors:** Sinisa Ducic, Stefan Djordjevic, Mikan Lazovic, Polina Pavicevic, Milena Mihajlovic, Amela Kalac, Filip Milanovic

**Affiliations:** 1 Pediatric Orthopedic Surgery Department, University Children’s Hospital, 11000 Belgrade, Serbia; sinisa.ducic@udk.bg.ac.rs (S.D.); mikan.lazovic@udk.bg.ac.rs (M.L.); 2 Faculty of Medicine, University of Belgrade, 11000 Belgrade, Serbia; stefan.djordjevic@udk.bg.ac.rs (S.D.); polina.pavicevic@udk.bg.ac.rs (P.P.); milena.mihajlovic@med.bg.ac.rs (M.M.); 3 Pediatric Deparment, University Children’s Hospital, 11000 Belgrade, Serbia; 4 Radiology Department, University Children’s Hospital, 11000 Belgrade, Serbia; 5 Institute of Pathology, Faculty of Medicine, University of Belgrade, 11000 Belgrade, Serbia; 6 Children’s Hospital, Clinical Center of Montenegro, 81000 Podgorica, Montenegro; amela.kalac@kccg.me

**Keywords:** *Bartonella henselae*, cat-scratch disease, pediatric osteomyelitis, case report, disseminated cat-scratch disease, multifocal osteomyelitis

## Abstract

**Highlights:**

**What are the main findings?**

- This case report highlights a rare presentation of multifocal pediatric osteomyelitis caused by *Bartonella henselae*.
- A multidisciplinary diagnostic approach, including imaging, serology, molecular testing, and histopathological evaluation, enabled the correct diagnosis.

**What is the implication of the main findings?**

- *Bartonella henselae* infection should be considered in the differential diagnosis of children with atypical multifocal bone lesions.
- Early recognition may prevent unnecessary invasive diagnostic procedures and inappropriate treatment, resulting in improved patient outcomes.

**Abstract:**

**Background/Objective**: *Bartonella henselae*, the causative agent of cat-scratch disease (CSD), usually causes a self-limiting zoonotic infection. Osteomyelitis is a rare but increasingly recognized manifestation, particularly in children, and may closely mimic malignant or other infectious bone diseases. We present a case of multifocal pediatric *Bartonella henselae* osteomyelitis and discuss the associated diagnostic and therapeutic challenges in the context of the current literature. **Case presentation**: We report the case of a previously healthy 10-year-old girl who presented with a one-month history of fever, fatigue, limping, and progressive thigh and groin pain following cat-scratch exposure. During the course of the illness, she developed unilateral facial nerve palsy and Parinaud oculoglandular syndrome. Initial laboratory investigations demonstrated elevated inflammatory markers and reactive thrombocytosis. Serological testing for *Bartonella henselae* was positive for both IgM and IgG antibodies, whereas polymerase chain reaction (PCR) testing of both peripheral blood and the bone biopsy specimen was negative. Plain radiographs were unremarkable; however, magnetic resonance imaging (MRI) revealed multifocal lesions involving the right femoral diaphysis, right pubic bone, and left iliac wing, together with focal hepatic and splenic lesions suggestive of disseminated disease. Because of the radiological suspicion of malignancy, a femoral bone biopsy was performed and demonstrated osteomyelitis with necrosis and microabscess formation, without evidence of malignancy. Following targeted antimicrobial therapy, the patient showed rapid clinical and laboratory improvement, with progressive regression of the lesions on follow-up MRI. **Conclusions**: *Bartonella henselae* osteomyelitis should be considered in the differential diagnosis of multifocal bone lesions in children, particularly in patients with a history of cat exposure. Because the disease may closely mimic malignancy both clinically and radiologically, establishing the diagnosis requires careful integration of epidemiological, clinical, serological, radiological, molecular, and histopathological findings. Early recognition and appropriate antimicrobial treatment are associated with an excellent prognosis.

## 1. Introduction

Cat-scratch disease (CSD) is a zoonotic infection caused by *Bartonella henselae*, a Gram-negative intracellular bacterium that is primarily transmitted through scratches or bites from infected cats. Until 1993, only three clinical syndromes had been attributed to *Bartonella* species: Carrion’s disease caused by *Bartonella baciliformis*, trench fever caused by *Bartonella quintana*, and cat-scratch disease caused by *Bartonella henselae*. Since then, the recognized clinical spectrum of Bartonella infections has expanded considerably [1,2,3,4].

Although CSD is generally a self-limiting disease characterized by a localized cutaneous lesion, regional lymphadenopathy, and involvement of the reticuloendothelial system, an increasing number of atypical manifestations have been described, particularly in immunocompromised individuals, although they may also occur in immunocompetent children [1,2,3,4,5,6]. Approximately 5–14% of patients develop systemic dissemination, with reported manifestations including prolonged fever, fatigue, myalgia, arthralgia, erythema nodosum, weight loss, granulomatous hepatitis, splenomegaly, and Parinaud oculoglandular syndrome. Neurological complications, such as encephalopathy and neuroretinitis, are less common but well recognized [4,6].

Musculoskeletal involvement is among the rarest complications of CSD, occurring in only a small proportion of infected children. Infection may involve both the axial and appendicular skeleton through hematogenous spread, lymphatic dissemination, or contiguous extension. Despite the increasing number of published reports, Bartonella osteomyelitis remains uncommon, and current evidence regarding its diagnosis and management is largely limited to case reports and small case series [6,7,8]. The vertebrae, pelvic girdle, ribs, and long bones are the most frequently affected skeletal sites, whereas prolonged fever, osteoarticular pain, and regional lymphadenopathy represent the most commonly reported clinical manifestations [6,7,9,10,11,12].

The diagnosis of Bartonella osteomyelitis remains challenging because both clinical presentation and imaging findings are non-specific. Radiographic evaluation, including plain radiography, magnetic resonance imaging (MRI), computed tomography (CT), and bone scintigraphy, may closely resemble malignant bone tumors, Langerhans cell histiocytosis, tuberculosis, chronic recurrent multifocal osteomyelitis (CRMO), or pyogenic osteomyelitis [6,9,13,14]. Conventional microbiological cultures are frequently negative, and diagnosis therefore relies primarily on serological testing, including indirect immunofluorescence assay (IFA) and enzyme immunoassay (EIA), with polymerase chain reaction (PCR) testing serving as an adjunctive diagnostic tool. However, due to the fastidious nature of the bacterium and the typically low bacterial burden, PCR results may also be negative, even in patients with confirmed disease [6,15,16,17,18,19,20]. Consequently, bone biopsy is often required, particularly when malignancy or alternative infectious etiologies cannot be excluded [6,7].

Although antimicrobial treatment for immunocompetent patients with Bartonella osteomyelitis remains controversial, several therapeutic regimens have been reported, including macrolides, rifampicin, doxycycline, trimethoprim-sulfamethoxazole, aminoglycosides, fluoroquinolones, and clindamycin [4,6,7].

Herein, we report a case of presumed multifocal *Bartonella henselae* osteomyelitis in an immunocompetent child, also presenting with hepatic and splenic involvement, Parinaud oculoglandular syndrome, and facial nerve palsy, with radiological findings highly suggestive of a malignant bone tumor. In addition to presenting this unusual case, we discuss the diagnostic challenges, differential diagnosis, and therapeutic considerations in light of the current literature.

## 2. Case Report

A previously healthy 10-year-old girl was referred to the University Children’s Hospital (UCH), Belgrade, Serbia, a tertiary pediatric referral center, because of a one-month history of persistent fever, fatigue, limping, reduced mobility, and progressively worsening thigh and groin pain. She had no significant past medical history. Further history revealed close contact with domestic cats, including a scratch injury to the face that had occurred seven days before symptom onset.

During the course of the illness, the patient developed atypical extracutaneous manifestations, including unilateral facial nerve palsy and Parinaud oculoglandular syndrome, characterized by unilateral granulomatous conjunctivitis and ipsilateral preauricular lymphadenopathy. Fever developed seven days after the cat scratch, followed by limping and thigh and groin pain on day 14, whereas facial nerve palsy and Parinaud oculoglandular syndrome appeared on day 20. She was subsequently admitted to our institution for further diagnostic evaluation after previously remaining hospitalized at the referring institution until transfer to our tertiary care center.

The patient initially received empirical antimicrobial therapy, consisting of intravenous ceftriaxone (50 mg/kg/day) from day 14 to day 20, followed by oral azithromycin (10 mg/kg/day) from day 20 to day 34. Initial laboratory investigations performed at the referring hospital demonstrated elevated inflammatory markers, including an erythrocyte sedimentation rate (ESR) of 80 mm/h and a C-reactive protein (CRP) level of 12.9 mg/L. Complete blood count revealed thrombocytosis, with a platelet count of 519 × 10^9^/L, whereas the leukocyte count remained within the reference range.

Serological testing for *Bartonella henselae* using an indirect immunofluorescence assay (IFA), performed on day 20, demonstrated positive qualitative IgM and IgG antibodies; however, antibody titers were not available. PCR analysis of peripheral blood, performed at our institution, was negative for *Bartonella henselae* DNA. The laboratory report did not specify the molecular target used for amplification. A Mantoux tuberculin skin test was also performed and yielded a negative result, making tuberculosis less likely.

**Figure 1:**
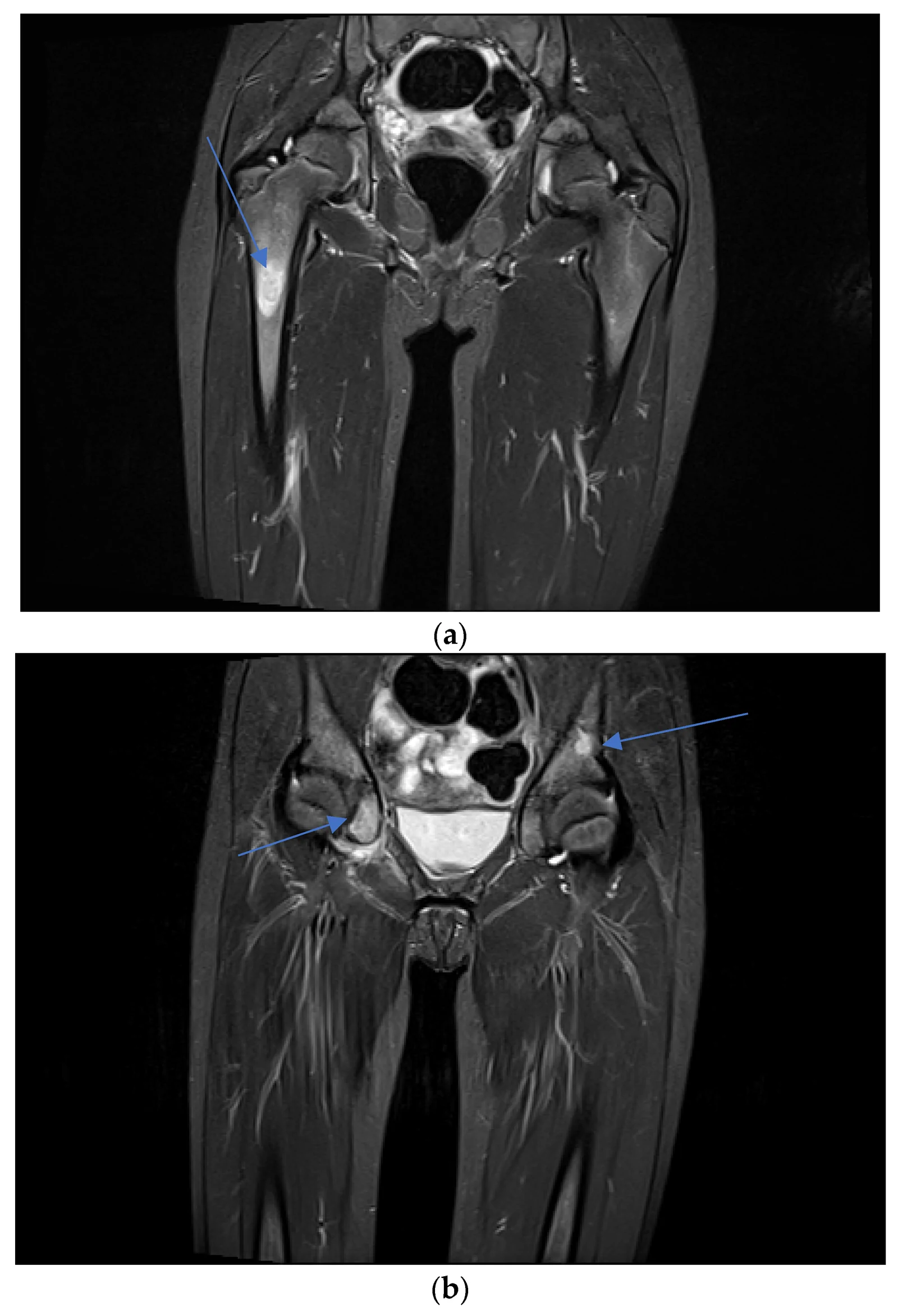
(**a**). Coronal T2-weighted MRI image showing a lesion of the right femur (arrow). (**b**). Coronal T2-weighted MRI image showing lesions of the right pubic bone and left iliac wing (arrows).

**Figure 2:**
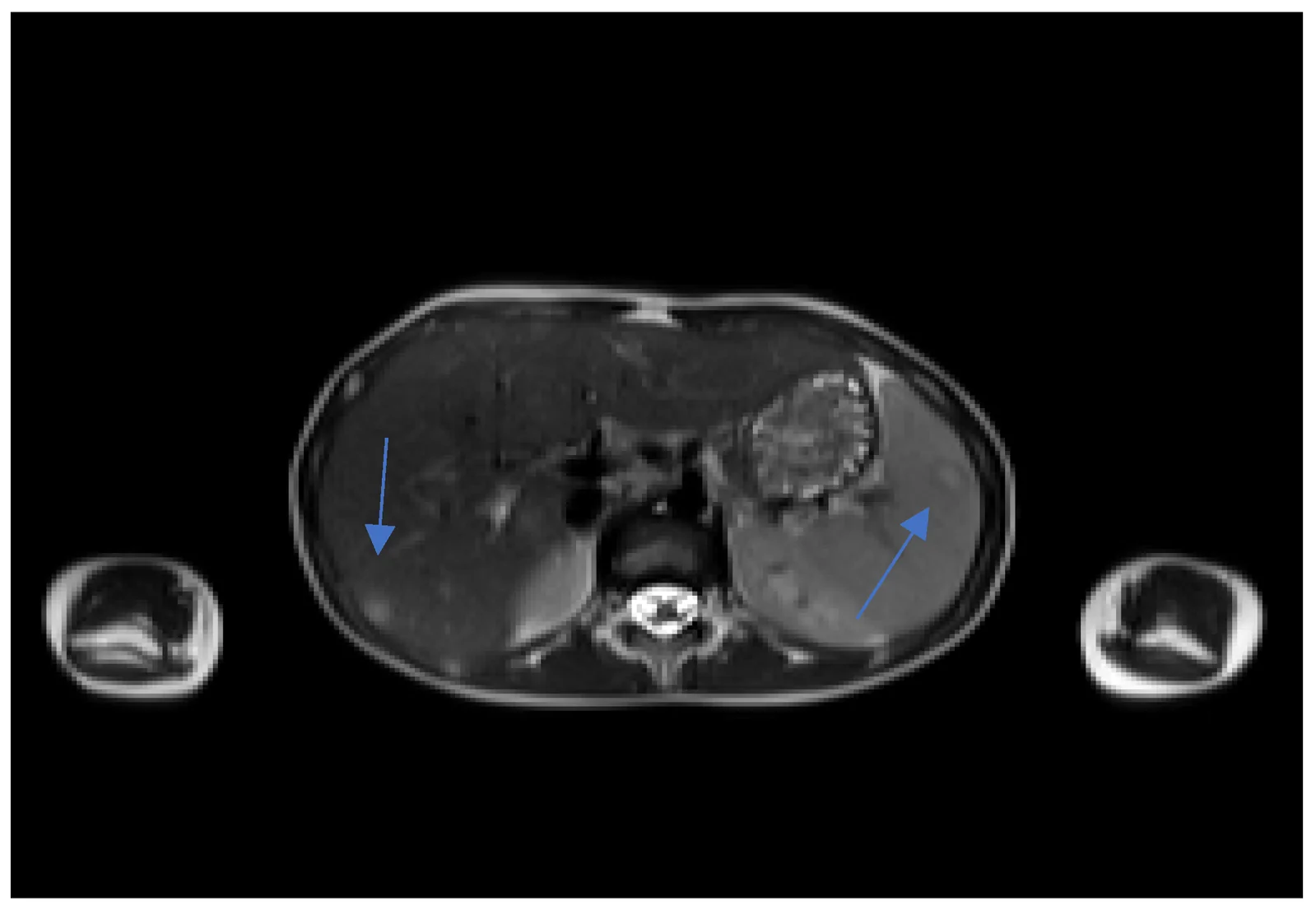
Axial T2-weighted MRI image showing focal hepatic and splenic lesions (arrows).

Plain radiographs showed no evidence of osseous abnormalities. However, MRI demonstrated multifocal bone lesions involving the right femoral diaphysis, right pubic bone, and left iliac wing, accompanied by mild edema of the right obturator externus and pectineus muscles (Figure 1). Whole-body MRI additionally revealed multiple focal lesions in the liver and spleen, consistent with disseminated disease (Figure 2).

Although positive *Bartonella henselae* serology supported the presumed diagnosis, the combination of multifocal skeletal lesions, adjacent soft-tissue involvement, and hepatosplenic lesions raised substantial concern for an underlying malignant process. Therefore, to exclude malignancy and establish a definitive tissue diagnosis, an open biopsy of the right femoral lesion, the most accessible site, was performed.

**Figure 3:**
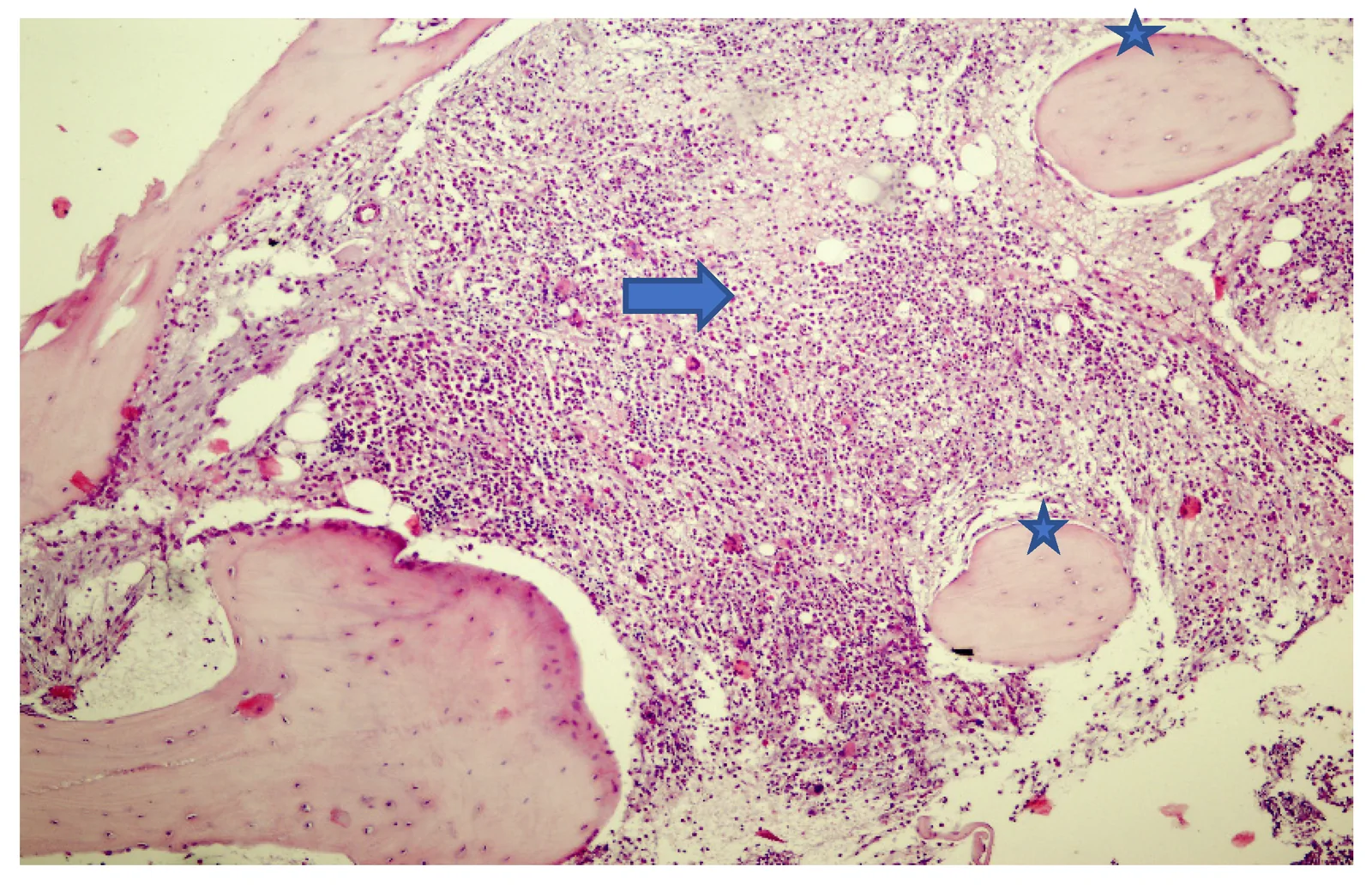
Histological image of bone tissue on an HE-stained sample, demonstrating areas of necrosis with abscess formation. Granulomas or malignant cells were not noticed (arrow indicates microabscess formation, whereas asterisks indicate necrotic bone trabeculae).

Histopathological examination demonstrated osteomyelitis characterized by extensive areas of necrosis and microabscess formation, without evidence of malignancy or granulomatous inflammation (Figure 3). PCR testing performed on the bone biopsy specimen was likewise negative for *Bartonella henselae* DNA. The molecular target of the assay was not specified in the laboratory report. Routine bacterial cultures showed no growth, fungal cultures were negative, and Warthin–Starry staining was not performed because of technical limitations.

Given the overall epidemiological, clinical, serological, and histopathological findings, together with exclusion of alternative diagnoses, the patient was considered to have presumed multifocal *Bartonella henselae* osteomyelitis. Empirical antimicrobial therapy was therefore replaced with targeted treatment, consisting of rifampicin (10 mg/kg/day) and doxycycline (6.7 mg/kg/day), following consultations with a pediatric infectious disease specialist and a review of the available literature.

The patient’s clinical condition improved rapidly after initiation of targeted therapy, accompanied by a progressive decline in inflammatory markers. Because of the potential hepatotoxicity of rifampicin, liver function tests were monitored every seven days and remained within the reference range throughout treatment. No treatment-related adverse events were observed.

The patient was discharged on day 44 to continue outpatient therapy, with regular clinical and laboratory follow-up. At the completion of the 12-week treatment course, she had achieved complete clinical recovery, normalization of inflammatory markers, and significant regression of all lesions on follow-up MRI.

The clinical course, diagnostic procedures, treatment, and follow-up are precisely shown in Table 1.

## 3. Discussion

Osteomyelitis is an uncommon manifestation of CSD, and its rarity frequently results in delayed diagnosis and extensive investigations aimed at excluding malignant and other infectious conditions [6,7,18,21,22]. Salmon-Rosseau et al. reported that osteomyelitis occurs in approximately 0.2–0.3% of *Bartonella henselae* infections, with nearly 80% of cases affecting children [12].

The clinical presentation of our patient was consistent with previously reported cases of Bartonella-associated osteomyelitis. Similarly to earlier reports, the disease occurred in an immunocompetent child with documented cat exposure and was characterized by elevated inflammatory markers, despite relatively non-specific clinical findings [3,7,22]. Notably, the marked elevation of ESR in the presence of only mildly elevated CRP levels, together with reactive thrombocytosis, suggested a subacute inflammatory process rather than acute pyogenic osteomyelitis.

Radiological evaluation played a pivotal role in the diagnostic work-up but was not sufficiently specific to establish the diagnosis. MRI demonstrated multifocal skeletal lesions associated with adjacent soft-tissue edema, findings that substantially overlap with those observed in Ewing sarcoma, Langerhans cell histiocytosis, CRMO, tuberculosis, and pyogenic osteomyelitis [22,23]. Consequently, imaging findings alone were insufficient to distinguish between infectious and malignant etiologies.

The differential diagnosis in our patient was particularly challenging because the combination of multifocal osteolytic bone lesions, prolonged fever, elevated inflammatory markers, and hepatosplenic lesions closely resembled both malignant and infectious diseases [6,9,14,22,23,24,25]. Ewing sarcoma and osteosarcoma were initially considered because of the radiological appearance of the lesions, but they were excluded by histopathological examination. Langerhans cell histiocytosis was also considered but was not supported by the pathological findings [24]. Tuberculosis represented another important differential diagnosis owing to the multifocal skeletal involvement. However, it was considered unlikely because of the negative tuberculin skin test, the absence of radiological features suggestive of tuberculosis, and the histopathological findings. CRMO was likewise considered, but the history of cat exposure, along with positive *Bartonella henselae* serology, hepatosplenic involvement, and the marked clinical response to targeted antimicrobial therapy strongly favored disseminated Bartonella infection.

Taken together, the epidemiological history, characteristic clinical manifestations, compatible imaging findings, positive serological results, histopathological exclusion of malignancy, and favorable response to targeted antimicrobial therapy provided strong evidence supporting the diagnosis of presumed *Bartonella henselae* osteomyelitis. Interestingly, granulomatous inflammation, which is frequently described in histopathological specimens from patients with Bartonella infection, was absent in our patient. Nevertheless, previous studies have demonstrated that granulomas may be absent during the early inflammatory phase of the disease and that their absence does not exclude the diagnosis [10].

Microbiological confirmation of *Bartonella henselae* infection remains challenging because the bacterium is fastidious and rarely identified by conventional culture techniques [7,16]. Consequently, serological testing remains the cornerstone of diagnosis, whereas PCR might provide additional evidence in selected patients [4,6,7,18]. However, negative PCR results do not exclude the infection. Mendez et al. recently reported negative PCR findings in approximately 71% of bone specimens obtained from children with Bartonella-associated osteomyelitis [26], emphasizing the limited sensitivity of molecular testing in this clinical setting.

Therapeutic recommendations for Bartonella osteomyelitis remain largely based on observational studies and published case reports because randomized controlled trials are lacking. Although spontaneous resolution has been described, antimicrobial therapy is generally recommended for patients with osteomyelitis or disseminated disease [4,6,7]. Macrolides, rifampicin, doxycycline, trimethoprim-sulfamethoxazole, aminoglycosides, fluoroquinolones, clindamycin, and combination regimens have all been associated with favorable clinical outcomes [4,6,7,14,23]. Our patient demonstrated rapid clinical improvement after initiation of rifampicin combined with doxycycline, a regimen that has also been successfully reported by Kopsidas et al. [27]. Furthermore, Lemos et al. emphasized that antimicrobial treatment should be individualized according to the clinical presentation and severity of bartonellosis [28]. More recently, Pizzuti et al. demonstrated that combination therapy with rifampicin and doxycycline represents one of the most frequently used treatment strategies for invasive Bartonella infections, often requiring prolonged treatment courses beyond traditionally cited six weeks [29].

The principal strength of this report lies in the comprehensive diagnostic evaluation, which combined epidemiological history, advanced imaging procedures, serology, PCR testing, histopathological examination, and longitudinal follow-up. Furthermore, the coexistence of multifocal osteomyelitis, hepatosplenic lesions, Parinaud oculoglandular syndrome, and facial nerve palsy represents an uncommon clinical presentation that may easily be mistaken for a malignant disorder.

Several limitations should also be acknowledged. Definitive microbiological confirmation was not achieved because PCR testing of both peripheral blood and bone tissue, as well as conventional bacterial cultures, yielded negative results. In addition, Warthin–Starry staining could not be performed because of technical limitations, and quantitative serological titers were unavailable. Consequently, the diagnosis should be regarded as presumed multifocal *Bartonella henselae* osteomyelitis, established through careful integration of epidemiological, clinical, radiological, serological, histopathological, and therapeutic findings.

Overall, this case underscores the importance of maintaining a high index of suspicion for disseminated *Bartonella henselae* infection in children presenting with multifocal bone lesions and systemic manifestations. Early recognition and a multidisciplinary diagnostic approach may facilitate timely diagnosis and lead to favorable clinical outcomes.

## 4. Conclusions

*Bartonella henselae* osteomyelitis should be considered in the differential diagnosis of multifocal bone lesions in children, particularly in the presence of a history of cat exposure and subacute clinical course. Because the disease may closely mimic malignant bone tumors both clinically and radiologically, establishing the diagnosis requires careful integration of epidemiological, clinical, radiological, serological, and histopathological findings. Although definitive microbiological confirmation may not always be achievable, a multidisciplinary approach can facilitate timely diagnosis and appropriate treatment. Early recognition and targeted antimicrobial therapy are associated with excellent clinical outcomes and favorable MRI resolution.

## Figures and Tables

**Table 1:** Timeline table of the clinical course, diagnostic procedures, treatment, and follow-up.

Timeline	Events	Investigations	Treatment
Day 0	Cat scratch	/	/
Day 7	Fever	/	/
Day 11	Fever	CBC, CRP, ESR	/
Day 13	Limping and thigh and groin pain	/	/
Day 14	Limping and thigh and groin pain	/	Ceftriaxone
Day 20	Facial nerve palsy and Parinaud oculoglandular syndrome	Serology	Azithromycin
Day 30	Admission to our hospital	CBC, CRP, ESR	Azithromycin
Day 31	Persistence of symptoms	PCR (blood sample)	Azithromycin
Day 33	Persistence of symptoms	MRI	Azithromycin
Day 34	Persistence of symptoms	Bone biopsy and PCR (bone specimen)	Rifampicin + doxycycline (for next 12 weeks)
Day 44	Progressive, daily clinical improvement, and discharge	Gradual and serial decline in inflammatory markers	The same therapy regimen
Day 44—3 months	Complete resolution of clinical symptoms during regular outpatient follow-up after discharge	Serial laboratory decrease in CRP and ESR, follow-up MRI regression of lesions	Off antimicrobial therapy after a full twelve-week treatment course

## Data Availability

All data supporting the findings of this case report are included within the article. Further data are available from the corresponding author upon reasonable request.
